# Random forest algorithm to identify factors associated with sports-related dental injuries in 6 to 13-year-old athlete children in Hamadan, Iran-2018 -a cross-sectional study

**DOI:** 10.1186/s13102-020-00217-5

**Published:** 2020-11-11

**Authors:** Maryam Farhadian, Sima Torkaman, Farzad Mojarad

**Affiliations:** 1 Department of Biostatistics, School of Public Health and Research Center for Health Sciences, Hamadan University of Medical Sciences, Hamadan, Iran; 2 Pediatric Dentistry Department, Dentistry School, Hamadan University of Medical Sciences, Hamadan, Iran; 3 Pediatric Dentistry Department, Dentistry School, Hamadan University of Medical Sciences, P.O. Box 4171-65175, Hamadan, Iran

**Keywords:** Sports-related dental injuries, Mouthguard, Random Forest, Athlete, Logistic regression

## Abstract

**Background:**

Traumatic dental injuries are one of the most important problems with major physical, aesthetic, psychological, social, functional and therapeutic problems that adversely affect the quality of life of children and adolescents. Recently the development of methods based on machine learning algorithms has provided researchers with more powerful tools to more accurate prediction in different domains and evaluate the factors affecting different phenomena more reliably than traditional regression models. This study tries to investigate the performance of random forest (RF) in identifying factors associated with sports-related dental injuries. Also, the accuracy of the RF model for predicting sports-related dental injuries was compared with logistic regression model as traditional competitor.

**Methods:**

This cross-sectional study was applied to 356 athlete children aged 6 to 13-year-old in Hamadan, Iran. Random forest and logistic regression constructed by using sports-related dental injuries as response variables and age, sex, parent’s education, child’s birth order, type of sports activity, duration of sports activity, awareness regarding the mouthguard, mouthguard use as input. A self-reported questionnaire was used to obtain information.

**Results:**

Fifty-five (15.4%) subjects had experienced a sports-related dental injury. The mean age of children with sports injuries was significantly higher than children without the experience of injury (*p* = 0.006). The prevalence of injury was significantly higher in boys (*p* = 0.008). Children with illiterate mothers are more likely to be injured than children with educated mothers (*p* = 0.045). Awareness of mouthguard and its use during exercise has a significant effect on reducing the prevalence of injury among users (*p* < 0.001).

Random forest model has a higher prediction accuracy (89.3%) for predicting sports-related dental injuries compared to the logistic regression (84.2%). The results of the relative importance of variables, based on RF showed, mouthguard use, and mouthguard awareness has more contributed importance in dental sport-related injuries’ prediction. Subsequently, the importance of sex and age is in the next position.

**Conclusions:**

Using predictive models such as RF challenges existing inaccurate predictions due to high complexity and interactions between variables would be minimized. This helps to achieve more accurate identification of factors in sport-related dental injury among the general population of children.

## Background

Traumatic dental injuries are one of the most important problems in oral health in children and adolescents. In addition to the physical aspect, it also impacts on psychosocial development through aesthetic concerns. These injuries can lead to impaired oral functions such as chewing and speech through severe dental or periodontal injuries such as tooth fracture, loosening, and direct erosion. Therefore, trauma to anterior teeth with major aesthetic, psychological, social, functional and therapeutic problems adversely affects one’s quality of life. Some part of the annual sport’s costs is spent on the treatment of sports-related dental injuries [1–5]. The cause of most dental injuries in children is their inability to identify traumatic situations. Traumatic dental injuries can occur not only during competitions but also during training and exercise sessions [6–8]. Almost 40% of dental injuries occur during sports activities [9].

Studies in different countries report different rates for tooth injuries in children. However, in a recent meta-analysis, the prevalence of dental injuries in children and adolescents worldwide is 17.5% and in boys twice as high as in girls [7].

Increasing numbers of violence, access to potentially risky recreational facilities, driving accidents, and greater participation of children in sports activities dramatically increased the dental trauma, making it an emerging public health problem [10].

Crashes, fights, sports, accidents, hitting objects or people are also factors that can cause tooth damage. The home, school, and street environments are the places most affected by tooth damage, most notably enamel fractures and dentin without pulp exposure [10–17].

Considerable research has also been done on the pathogenic, predisposing and risk factors for such injuries. Based on the available evidence, these factors can be broadly categorized into anatomical and social-behavioral factors. Anatomical factors that increase the risk of anterior tooth injuries include maxillary incisor overjet and teeth inadequate lip coverage of the anterior [11, 12]. Predictors of social behavioral factors also include sex, adverse social-psychological environment, problematic behavior, increased participation in sports, recreational activities and accidents [9, 11].

Therefore, identifying the factors associated with the prevalence of sports-related dental injuries in children is an important step in preventing them and will promote the oral health of future athletes.

The most previous research for identifying the factors associated with sports-related dental injuries is widely employed descriptive statistics methods and classical models such as the logistic regression model. However, in recent years the development of methods based on machine learning algorithms which account for non-linear relationships has provided researchers with more powerful tools to more accurate predictions in different domains and evaluate the factors affecting different phenomena more reliably. There are several supervised learning algorithms try to model relationships and providing acceptable classification models [17, 18].

Decision-tree algorithms such as random forest (RF), because of simplicity, are more popular than other machine learning algorithms in a different area [19]. Decision trees are constructed through a sequential separation of data into distinct groups, and the purpose of this process is to increase the distance between groups in each isolation. One of the differences between decision tree methods is how this distance is measured. RF is a Tree-based method in the field of machine learning for classification and regression purposes. The RF is a supervised learning method that ultimately leads to a simple understanding and interpretation of its results by the user. Also, the production of prediction rules is a feature of the RF method. Prediction rules are logical statements of the form if (conditions) then (prediction) which are easy to use in decision making [20, 21].

Given these promising features, this study tries to investigate the performance of RF in identification factors associated with sports-related dental injuries in 6 to 13-year-old athlete children in Hamadan west of Iran. In this study, RF will be used for predicting sports-related dental injuries. Also, the relative importance of variables in the prediction of sports-related dental injuries will be identified. In this way, the accuracy of the RF model for predicting sports-related dental injuries was compared with logistic regression model as traditional competitor.

## Methods

### Ethical approval and consent to participate

The study was approved by a research ethics committee of Hamadan University of Medical Sciences with IR.UMSHA.REC.1397. 728 codes.

### Participants

This cross-sectional study was carried out using a multi-stage cluster sampling method with randomly selected 356 athlete children aged 6–13 years who are active in sports clubs in Hamadan city (west of Iran) and also have more than 1 year of sports experience.

The sample size was calculated based on a sample error of 0.05, a significance level of 5%, and the prevalence of dental injuries of 20% and the design effects of 1.5. With a response rate of 70%, finally, 356 questionnaires were used for analysis.

All the clubs in the city included in the sampling frame, the clubs served as clusters, after random selection of clubs, athlete children were randomly selected in each sport. Those children who belonged to multiple sports clubs were excluded from this study. A letter was sent to all parents or guardians of the selected children explaining the purpose, characteristics, and importance of the study. All athlete children that the parent or guardian provided informed consent on behalf of the child were included in this study. Eligible participants were identified and information collected from June to October 2018.

### Data collection

A self-reported questionnaire was used to obtain information on the sports-related dental injury. The questionnaire of this study was designed based on similar studies and literature reviews [9, 13, 22, 23]. The questionnaire was divided into three sections. The first part consisted of questions related to age, sex, parental education, child’s birth order, type and history of exercise activity, duration of exercise activity during the week and day and enjoyment of playing. The second part included questions about the history of dental injury, the time of injury, the type of dental injury, the time of referral to medical centers. The third section also included questions about athlete awareness and use of oral protective equipment such as mouthguards [24]. To assess the validity of the developed questionnaire face and content validity was used. Also, Cronbach’s alpha coefficient assessed internal consistency. After confirming the reliability and validity, the questionnaire sent to parents.

The parents’ response to the question “Has your child ever had a tooth injury during exercise” was used to assess the prevalence of dental injuries during exercise. Type of activity depending on exposure was divided into the non-contact sport: gymnastics, limited-contact sport: involving football, volleyball and basketball, semi-contact sport: karate and taekwondo, and full-contact sport including wrestling, boxing, and judo. The type of tooth injury was divided into types of crown fractures, mobility, and complete tooth extraction so that parents can be understood.

### Statistical analysis

#### Descriptive and bivariate analysis

To summarize categorical study variables frequencies and percentages were used, and mean and standard deviations were computed for continuous variables. Furthermore, the univariate association of dental injury with categorical variables was analyzed by the Chi-square test. The significance level was considered to be 0.05. The analysis was performed using SPSS 21 software.

#### Random forest

The RF algorithm is a recursive partitioning method generates large amounts of trees and then averages the results. Initially, bootstrap data sets were created through the resampling of the training data. Then for each of the bootstrap samples, RF will construct an unpruned tree according to the following procedure: at each node of the tree number of the predictors randomly selected and then selects the best split among all predictors. The classification error rate of the RF, which so-called out of bag (OOB) error will be estimated by considering all excluded samples by bootstrap samples. Finally, the one final classification is consists of the outputs of all trees [19, 20].

In this study, RF constructed by using sports-related dental injuries as response variables (including 2 class label: yes and no) and age, sex, parents education, child’s birth order, type of sports activity, duration of sports activity, awareness regarding the mouth guard, mouth guard use, are used as input as predictor variables.

### Variable importance

The output of the variable importance is one of the main features of RF. Variable importance describes the relationship between a given variable and the classification result. In this regard, the permutation importance index was used in this study to assess variable importance. Calculation of the variable importance is performed by looking at the change in prediction error occurring when OOB data for that variable is randomly permuted while all other variables are left unchanged. The calculations are performed tree by tree while the RF is drawn. Compared to variables that are not important, permuting values of an important variable in the analysis problem at random leads to greater changes in prediction performance [19, 20].

We used default parameters for RF: the number of trees (ntree) equal to 1000 and the number of variables analyzed at each node to find the best split where the total number of variables in the problem is. Statistical analyses were performed using R packages random Forest and caret.

### Logistic regression

A logistic regression model was also used to evaluate the impact of different factors on dental sports injuries. It should be noted that the independents and dependent variables in the logistics regression model were similar to the random forest model. The results were presented in terms of odds ratio and 95% confidence interval for the odds ratio.

### Model evaluation

The predictive performance of random forest and logistic regression models are evaluated by constructing the confusion matrix. Besides, accuracy is also measured for each model.

## Results

Characteristics of the subjects according to the sports-related dental injury presented in Table 1. According to the results, of 356 participating children, 55 (15.4%) subjects experienced sports-related dental injury and 301 subjects (84.6%) had no history of sports-related dental injury. The mean age of children with sports injury (11.31 ± 1.61 years) was significantly higher than children without the experience of injury (10.61 ± 2.14 years) (*p* = 0.006). According to the univariate analysis based on the Chi-square test, the prevalence of injury was significantly higher in boys (20.1%) than in girls (9.9%) (*p* = 0.008). A mother’s level of education has a significant effect on the prevalence of dental sport-related injury (*p* = 0.045). The injury was higher in children who had first child than other children, although this difference was not significant (*p* = 0.407). Among the children with sports-related dental injuries, 36.4% (*n* = 20) had crown fracture, 58% (*n* = 32) had mobility and 5.6% (*n* = 3) had avulsion. There is no significant difference in the prevalence of injury in terms of experience and duration of exercise per week and day (*p* > 0.05). Awareness of mouthguard and its use during exercise has a significant effect on reducing the prevalence of injury among users (*p* < 0.001). Only 7.7% of people who have knowledge about the mouthguard has been injured, while 23.7% of people who were unaware suffered from dental injury (*p* < 0.001). The prevalence of injury was significantly lower among users of a mouthguard (7.8%) than the non-users of a mouthguard (17.6%) (*p* < 0.001).

**Table 1:** Characteristics of the subjects according to the sports-related dental injury

Variable	Sports related dental injury				Total	
	Yes		No			
	N	%	N	%	N	%
**All**	55	15.4	301	84.6	356	100
Age (year): Mean ± SD	11.31 ± 1.61		10.61 ± 2.14		*P*-value*: 0.006	
Sex					N	*P*-value**
Boy	155	79.9	39	20.1	194	0.008
Girl	146	99.1	16	9.9	162	
Father Education						
Diploma	164	87.2	24	12.8	188	0.055
Bachelor	104	78.8	28	21.2	132	
PhD	33	91.7	3	8.3	36	
Mother Education						
Illiterate	12	79.6	5	29.4	17	0.045
Diploma	173	87.7	24	12.2	197	
Bachelor	91	79.1	24	20.9	115	
PhD	25	92.6	2	7.4	27	
Child’s Birth Order						
**1**	160	82.9	33	17.1	193	0.407
2	116	85.3	20	14.7	136	
3	25	92.6	2	7.4	27	
Sport						
Non-Contact	32	94.1	2	5.9	34	0.025
Limited-Contact	55	82.1	12	17.2	65	
Semi-Contact	61	93.8	4	6.2	67	
Full-Contact	153	80.5	37	19.5	190	
Experience [years]						
1–5	276	84.9	49	15.1	325	0.529
> 5	25	80.6	6	19.4	31	
Training days per week						
< 3	135	88.2	18	11.8	153	0.095
> 3	166	81.8	37	18.2	203	
Training hour per day						
< 3	188	86.6	29	13.4	217	0.266
3–5	87	79.8	22	20.2	109	
> =5	25	86.2	4	13.8	22	
Mouth guard awareness						
No	132	76.3	41	23.7	173	< 0.001
Yes	169	92.3	14	7.7	183	
Mouth guard Use						
No	230	82.4	49	17.6	279	< 0.001
Yes	71	92.2	6	7.8	77	
Type of injury						
Crown fracture	20	36.4				
mobility	32	58				
avulsion	3	5.6				

* T-Test **Chi square test

Based on the results of multiple logistic regression model presented in Table 2, increasing age significantly increases the chance of injury occurring, with a one-year increase in age approximately 1.3 times the odds of injury was increased (95%CI: 1.04–1.55, *p* = 0.021). The odds of injury in boys are 2.3 times higher than girls (95%CI: 1.05–5.04, *p* = 0.037). Children that no awareness about mouth guard had 5.44 times more likely to having a dental injury than those with the awareness about a mouthguard (95%CI: 2.51–11.8, *p* < 0.001). Also, the odds of injury to those who did not use the mouth guard is approximately 9 times higher than those who did use the mouthguard during exercise (95%CI: 3.22–21.6, *p* < 0.001).

**Table 2:** Logistic regression analyses of factors associated with a sport-related dental injury

Variable	Unadjusted odds ratio	95% CI	***P***-value	Adjusted odds ratio	95% CI	***P***-value
Age (year)	1.21	1.02–1.41	0.024	1.27	1.04–1.55	0.021
Sex			0.009			0.037
Girl	Reference			Reference		
Boy	2.29	1.23–4.29		2.31	1.05–5.04	
Father’s education			0.061			0.272
PhD	Reference			Reference		
Bachelor	2.96	0.85–10.67	0.090	2.84	0.45–18.14	0.312
Diploma	1.61	0.46–5.66	0.458	2.11	0.49–8.95	0.845
Mother’s education			0.053			0.104
PhD	Reference			Reference		
Bachelor	3.29	0.73–14.91	0.121	3.16	0.55–8.42	0.200
Diploma	1.73	0.39–7.79	0.473	2.84	0.45–18.14	0.270
Illiterate	5.21	0.88–30.83	0.069	12.19	1.32–82.33	0.027
Child’s Birth Order			0.426			0.430
3	Reference			Reference		
2	2.57	0.58–11.42	0.321	2.67	0.51–13.88	0.242
1	2.15	0.47–9.82	0.212	2.90	0.58–14.47	0.194
Sport			0.039			0.285
Non-Contact	Reference			Reference		
Limited-Contact	3.49	0.73–16.59	0.116	4.05	0.71–23.09	0.116
Semi-Contact	1.05	0.18–6.04	0.957	1.57	0.23–10.65	0.642
Full-Contact	3.76	0.89–16.88	0.072	3.21	0.63–16.28	0.159
Experience [years]			0.530			0.830
> 5	Reference			Reference		
1–5	0.74	0.28–1.89		0.719	0.26–2.55	
Training days per week			0.970			0.559
> 3	Reference			Reference		
< 3	0.59	0.33–1.09		0.795	0.37–1.72	
Training hour per day			0.264			0.757
> =5	Reference			Reference		
3–5	0.96	0.31–2.95	0.942	1.43	0.37–5.48	0.598
< 3	1.58	0.49–5.01	0.457	1.64	0.44–6.16	0.465
Mouth guard Awareness			< 0.001			< 0.001
Yes	Reference			Reference		
No	3.75	1.96–7.17		5.44	2.51–11.8	
Mouth guard Use			0.041			< 0.001
Yes	Reference			Reference		
No	2.52	1.04–6.13		8.93	3.22–21.6	

Also, the performance of both multiple logistic regression and random forest models in predicting dental sport-related injury was evaluated. The confusion matrix along with the accuracy of each model are provided in Table 3. The results showed that the random forest classification model has a higher prediction accuracy (89.3%) for sports-related dental injuries compared to the logistic regression model (84.2%). However, both models had less accuracy in predicting those who were injured than those who were not.

**Table 3:** Predictive performance measure of random forest and logistic regression models

		Random forest		Logistic	
		True class		True class	
		No	Yes	No	Yes
Predicted class	No	293	30	289	44
	Yes	8	25	12	11
Accuracy		0.893		0.842	

**Fig. 1:**
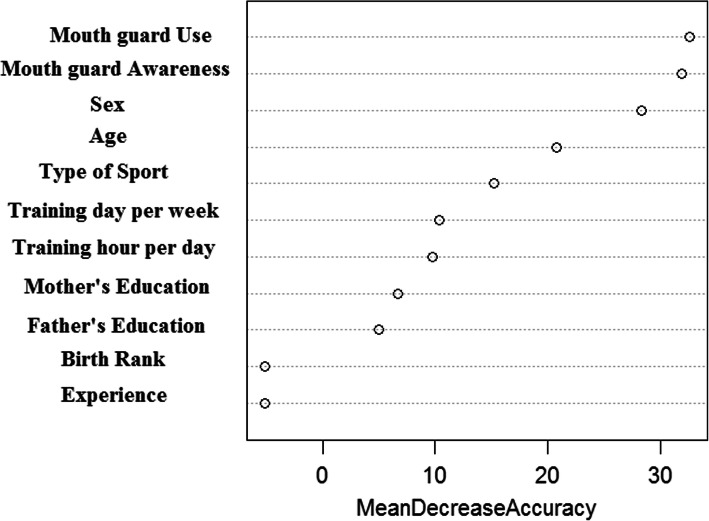
Variable importance of Random Forest model for predicting sports-related dental injuries in 6 to 13-year-old athlete children in Hamadan, Iran-2018

The results of the relative importance of each variable, based on the random forest model, in terms of mean decrease in accuracy, are presented in Fig. 1. The results showed mouthguard use, and mouth guard awareness has more contributed importance in dental sport-related injuries’ prediction. Subsequently, the importance of sex and age is in the next position.

## Discussion

In this study, the prevalence and factors affecting the sport-related dental injury were evaluated using logistic regression and random forest models. The results indicated that both models have a good prediction performance in terms of accuracy. However, the accuracy of the random forest model was better than the regression model. Also, the results of the variable importance based on the random forest model indicate that mouthguard use and mouth guard awareness have higher relative importance than other variables. Subsequently, sex and age were more contributed to the prediction of injury. These findings are consistent with those significantly variables identified in the multiple logistic regression model.

Our results show that the prevalence of sports-related dental injuries was 15.4%; this similar pattern is seen in similarly aged cohort of athletes from Japan with 13.3% prevalence [9]. In the study conducted by Rouhani et al. on 80 professional contact sports athletes aged 20–30 years in northeastern Iran, 26.2% of athletes experienced one type of dental injury [22]. However, the prevalence of dental injury in the study of Paiva et al. on 12-year-old of Brazilian children was 34.9% [23]. In the Singh et al., in high school students aged 8–16 in northern India, 32% of girls and 29% of boys had a sports injury [25].

According to the literature, male is at greater risk of sports-related dental injury. Boys are usually more active and engage in stronger physical activities such as contact sports, fights, harder games, and use toys and equipment with a higher risk potential without adequate protection [7]. In the present study, the incidence of injury in boys was twice that of girls.

In the absence of mouthguards, the risk of injury is 1.6–1.9 times higher, and several review studies have shown that using mouthguards was effective in reducing soft and hard tissue injuries [7, 26]. The mouthguards distribute the force of the blows to the mouth and reduce the damage. The results of the present study also confirm this issue, that way the use of mouthgraud reduces the risk of injury by approximately 2.5 times.

Intensity and frequency of contact are major contributors to these injuries. Higher risk of dental injuries happened in direct contact sports like boxing, soccer, basketball, and hockey [8]. The results of this study also showed that the chance of injury in contact sports is significantly higher than non-contact sport.

The most common type of traumatic injuries in the teeth is enamel fracture and consequently enamel and dentin fracture [15, 16]. In this study, crown fracture with 36.4% is one of the most type of injury.

Although some researchers have reported that high school students with lower socioeconomic status are more likely to develop sport-related dental injuries, there are inconsistencies in various studies in this area [6]. In the present study, children with illiterate mothers are more likely to be injured than children with educated mothers. This can be due to these children’s unfamiliarity with the mouthguards and even because these children play more contact sports.

With the development of machine learning models that are more predictive than conventional regression models, the need to use such models in a variety of contexts, including predicting and identifying factors affecting sports-related dental injury, has increased. Nowadays, random forest has been successfully applied for prediction and classification purposes in many scientific realms.

Although among all machine learning approaches RF represents valuable results in many scientific fields [27–29], it is still poorly applied in the context of sports dentistry and its related area. Even very limited studies have used decision tree-based algorithms in the field of dentistry. For example, Dima et al., applied the decision tree algorithm to investigate the effect of parental oral health on the experience of dental caries in children. The results showed that the model used in this study had an accuracy of 93.33% [30].

As mentioned, studies in the sports-related dental injuries area mainly use descriptive statistics and statistical tests such as chi-square and logistic regression models to analyze the results and identify factors affecting dental injury. However, none of these studies reported the ability to predict the regression model, therefore, it is not possible to compare the performance of these models with the random forest model in the present study.

### Limitation

One of the limitations of the present study, as a secondary study based on information from study aimed at assessing the prevalence of dental injuries and mouthgards use in children, is the lack of access to other important information such as social-behavioral and anatomical factors. Also, this study was performed as cross-sectional and the inverse causal relationship between exercise-related dental injury and study variables was not determined. In addition, the answers to the self-reported questions may have been influenced by the recall bias.

## Conclusion

Using predictive models such as random forest challenges existing inaccurate predictions due to high complexity and interactions between variables would be minimized. Such algorithms can be used to identify children at risk for sports-related dental injuries. This helps to achieve more accurate identification of factors in sport-related dental injury among the general population of children.

Increased awareness, the existence of laws to force the use of oral protective equipment in high-risk sports, and encouraging athletes to use oral protective equipment regularly can reduce the occurrence of dental injuries. Children, and especially their parents, should be informed about the risks of dental injuries and their aftermath and the benefits of using the proper type of oral protection.

## Data Availability

The datasets during and/or analyzed during the current study available from the corresponding author on reasonable request.

## Abbreviation

RF: Random forest
